# Kinetics of Carotenoids Degradation during the Storage of Encapsulated Carrot Waste Extracts

**DOI:** 10.3390/molecules27248759

**Published:** 2022-12-10

**Authors:** Vanja Šeregelj, Lorenzo Estivi, Andrea Brandolini, Gordana Ćetković, Vesna Tumbas Šaponjac, Alyssa Hidalgo

**Affiliations:** 1 Faculty of Technology, University of Novi Sad, Bulevar Cara Lazara 1, 21101 Novi Sad, Serbia; 2 Department of Food, Environmental and Nutritional Sciences (DeFENS), Università degli Studi di Milano, Via Celoria 2, 20133 Milan, Italy; 3 Council for Agricultural Research and Economics-Centre for Animal Production and Aquaculture (CREA-ZA), Viale Piacenza 29, 26900 Lodi, Italy

**Keywords:** activation energy, Arrhenius, β-carotene, freeze-drying encapsulation, spray-drying encapsulation

## Abstract

The encapsulates of carrot waste oil extract improved the antioxidant properties of durum wheat pasta. The aim of this research was to study the kinetics of carotenoids degradation in the freeze-dried (FDE) and spray-dried (SDE) encapsulates of carrot waste extract during storage at four different temperatures (+4, +21.3, +30, +37 °C) up to 413 days by HPLC. Carotenoids levels decreased as a function of time and temperature, following zero-order kinetics. At 4 °C carotenes were stable for at least 413 days, but their half-lives decreased with increasing temperatures: 8–12 months at 21 °C; 3–4 months at 30 °C; and 1.5–2 months at 37 °C. The freeze-drying technique was more effective against carotenes degradation. An initial lag-time with no or very limited carotenes degradation was observed: from one week at 37 °C up to 3 months (SDE) or more (FDE) at 21 °C. The activation energies (Ea) varied between 66.6 and 79.5 kJ/mol, and Ea values tended to be higher in FDE than in SDE.

## 1. Introduction

Carotenoids are widely distributed fat-soluble pigments, which naturally exhibit the red, orange, and yellow color of fruits and vegetables. Recent interest in carotenoids mostly arises from their health benefits. Some carotenoids serve as precursors of vitamin A. Vitamin A is essential for vision, reproduction, and bone health [1]. In addition, vitamin A plays an important role in the immune system function; the intake of recommended daily levels of this vitamin strengthens the host response to infection, which is also important in the context of the new infection COVID-19 [2]. Among others, scientific literature suggests an inverse correlation between the consumption of carotenoids and cancer incidence, cardiovascular diseases, oxidative stress, and eye-related diseases, etc. [3].

Increasing consumer awareness and legislative actions against the use of synthetic colorants pushed the food industry to create functional food products with natural pigments, such as carotenoids. However, food industry expansion leads to the production of a large amount of waste materials rich in valuable molecules, whose conversion into economically valuable goods is supported within the circular food processing concept. Carrot is among the 10 most important vegetable crops in the world. About 25–30% of globally produced carrots are aesthetically unsuitable for the fresh market and are transformed into juices, discarding 30–40% of raw material as exhausted pulp [4]. Carrot pomace is rich in valuable bioactive components, hence multiple studies tested its upcycle, going beyond the destination of waste for livestock feed [4,5]. 

The extraction of carotenoids with edible oils has been extensively studied [6,7]. This process has multiple advantages by using green, non-toxic, non-hazardous, and inexpensive solvents [8]. The oil solubilizes the carotenoids and facilitates their intestinal absorption, which requires the formation of micelles [9]. Therefore, a carotenoid-enriched oil appears as an excellent carrier, and also for the preparation of concentrated nutraceutical and functional ingredients. Conversely, in raw carrots, cell compartmentation hinders the release and absorption of carotenoids, which can be improved by disrupting the tissues, through cooking or puréeing, or by adding fat [10].

Besides poor bioavailability from native sources, carotenoids are very susceptible to degradation. In fact, the high number of conjugated double bonds makes carotenoids prone to autooxidation and isomerization during food processing and storage [11]. A promising technological strategy to improve their stability is encapsulation; among all encapsulation techniques, freeze-drying and spray-drying are commonly used methods for preserving carotenoids [12]. In the food industry, this technology is widely used to develop functional ingredients, protected against environmental stresses such as oxygen, temperature, light, pH, and free radicals. Additionally, the enrichment of durum wheat pasta with 10% or 20% encapsulated carotenoids significantly improved the nutritional quality of the product [13] without penalizing technological and sensorial qualities [14].

Some information on carotenoid oil extraction combined with encapsulation exists [4,8,15], but valuable data on the degradation kinetics during the storage of encapsulated carotenoids are scant. Thus, the aim of this study was to investigate the stability of carotenoids in encapsulated carrot waste extract prepared by freeze-drying and spray-drying techniques during storage at four different temperatures (+4, +21.3, +30, +37 °C).

## 2. Results and Discussion

The carotenoids found in the encapsulates were α-carotene, β-carotene, and cis-β-carotene. The initial carotenes content corresponded, respectively, to 5.5, 18.1, and 5.8 mg/kg DM for the spray-dried encapsulate (SDE) and to 5.5, 18.5 and 5.0 mg/kg DM for the freeze-dried encapsulate (FDE). The cis-β-carotenes likely derive from isomerization reactions of all-trans-β-carotene and are generally absent in fresh carrots [16] or very scarce (1% of total carotenoids) [17], although recently Fratianni et al. [18] discovered 10.5% cis-β-carotenes. The β-carotene isomerizes towards the cis form only if solubilized, and the extent of isomerization is linearly dependent on the temperature in the range 100–140 °C [16]. Qiu et al. [19] confirmed that solubilized β-carotene isomerizes more readily and that cis forms are thermodynamically less stable. Isomerization leads to a reduction in β-carotene activity as provitamin A: 13-cis-β-carotene has an activity of 53%, equal to that of all-trans-α-carotene, while 9-cis-β-carotene reaches only 38% [20]; besides, cis isomers are less bioavailable and less effective in quenching singlet oxygen than trans isomers [21]. In juice processing, carotenes can be solubilized by the small amounts of carrot lipids during a prolonged (60 min), high temperature (100 °C) blanching, which breaks down the cellular matrix [16,21]. Likely, this facilitates the isomerization: indeed, boiling carrots for 15 min increases 10-fold or more cis-β-carotene concentration [17]. Blanching, however, is necessary to inactivate pectin esterases and preserve the correct turbidity of carrot juice [22]; additionally, it improves the retention of β-carotene and reduces enzymatic browning in dried carrots [23]. Most of cis-β-carotene was likely derived from blanching, but a further slight increase may be due to the high temperature (130 °C) at which the SDE mixture was injected, or to the time the spray-dried powder spent in the hot collecting recipient [24].

**Figure 1:**
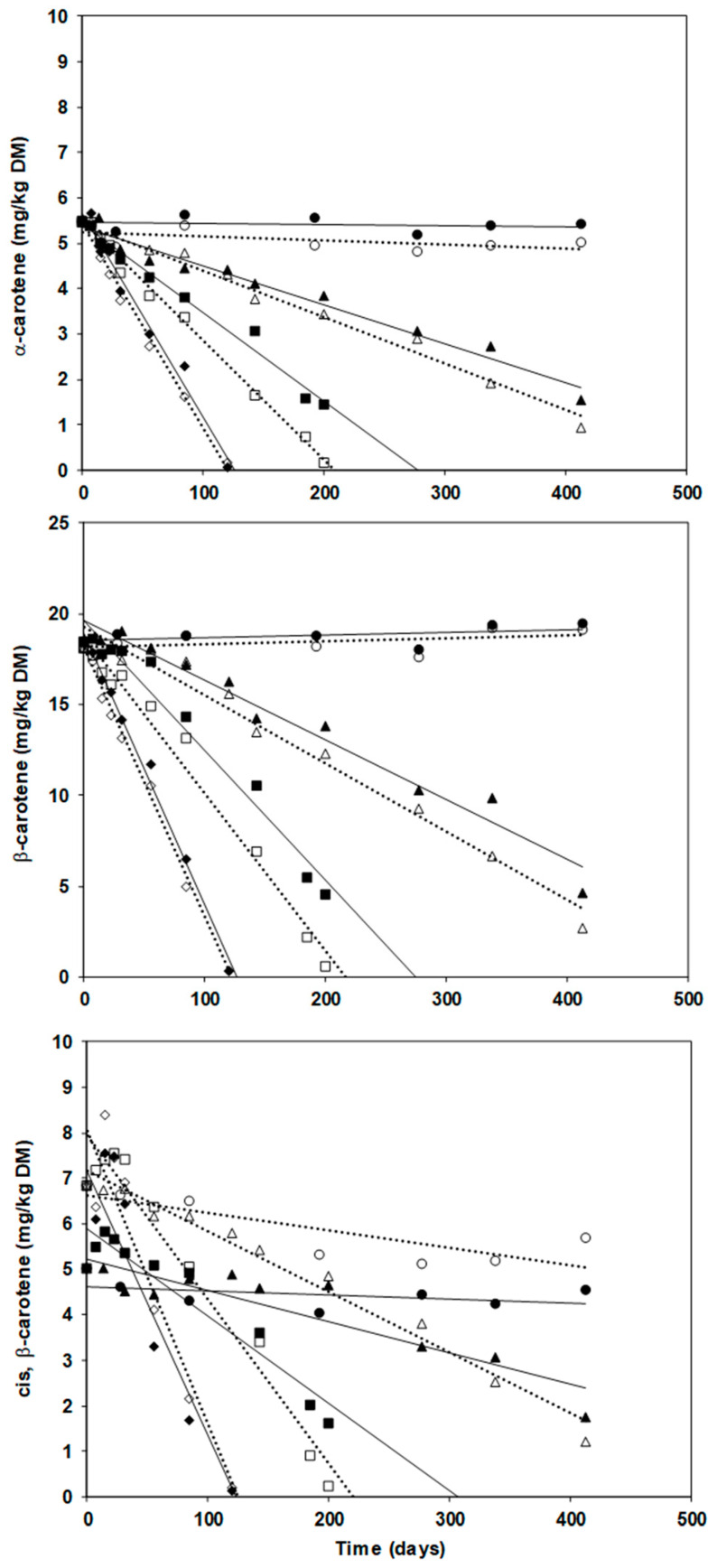
Degradation kinetics of α-carotene, β-carotene, and cis-β-carotene during storage at 4 °C (●, ○), 21.3 °C (▲, Δ), 30 °C (■, □) and 37 °C (♦, ◊) of freeze-dried (solid marks and solid regression lines) and spray-dried (hollow marks and dotted regression lines) encapsulated carrot-waste extract.

Figure 1 depicts the changes of carotenoids content in the encapsulates stored at 37, 30, 21.3, and 4 °C. 

Table 1 reports the regression coefficient of correlation (r) and the reaction rate constants of zero-order kinetics and first-order kinetics. The correlation suggested that a zero-order equation best modelled carotenoids degradation. Thus, the degradation reaction equation was C = C_0_ − kt, where C_0_ is the initial concentration, k is the reaction rate constant, t is the reaction time (days). The degradation rate depended on the temperature, but at 4 °C the carotenes were stable for at least 413 days. The cis-β-carotene exhibited an atypical pattern increasing at first, likely because of isomerization side reactions, and then decreasing for its degradation to epoxides, apocarotenones, and apocarotenals [11]. This behavior was observed also in dehydrated pumpkins stored at 4, 25 and 40 °C: the total cis isomers initially increased up to 30 days, but their growth was more evident where the headspace had been filled with nitrogen: this implies oxidative reactions prevail on isomerization whether oxygen is present [25].

The faster oxidation of the superficial layer, followed by delayed depletion of encapsulated carotenoids, likely induces a pseudo-first order kinetic of carotenoids degradation in dried preparations [26]. In fact, first order kinetics were reported for gac oil spray-dried encapsulated in gum Arabic and whey protein stored at −20 °C, 10 °C, room temperature, 40 °C and 63 °C [27]; for red palm oil spray-dried encapsulated in maltodextrin, sodium caseinate and soy lecithin, and stored at 25, 45, 65, and 85 °C [28]; and for norbixin encapsulated in different mixes of gum Arabic and maltodextrin and subject to storage accelerated conditions (60–98 °C, up to 300 min) [29]. However, zero-order kinetics were described as well for gac peel oil spray-dried encapsulated in whey protein concentrate and Arabic gum and stored at 5 and 20 °C for six months [30], and for sunflower oil enriched with pumpkin pomace carotenoids, spray-dried encapsulated in gum Arabic and stored at 25 °C for 90 days [31]. In addition, linear behaviors in carotenoids loss were observed during storage of β-carotene encapsulated in liquid emulsion through gelation of sodium alginate [32] or carrageenan [33].

Table 1 shows that the estimated half-lives decrease with increasing temperatures: 8–12 months at 21.3 °C, 3–4 months at 30 °C and 1.5–2 months at 37 °C. These values are similar to those (5–15 months) reported for α- and β-carotene extracted from carrots, encapsulated in partially hydrolysed starch, and stored at 21 °C [34].

Additionally, they fall within the range (5.5–87.2 weeks) of trans-β-carotene encapsulated in maltodextrins, variously added with glucose, galactose, or lactose, and stored at 25 °C [35]. Similarly, Lim et al. [36] encapsulated sunflower oil enriched with 0.05% β-carotene and lutein by using trehalose as wall material and noticed that 48–51% of β-carotene was retained at 25 °C and 36–41% at 37 °C. Przybysz et al. [37] spray-dried rapeseed oil enriched with carotenoids extracted from carrot juice in gum Arabic and maltodextrin; their encapsulate had a much higher total carotenes concentration than ours (20.6 mg/100 g), with an α:β-carotene ratio of 1:2. Without encapsulation the α- and β-carotene halved in 6 weeks, while encapsulated carotenes half-lives were approximately 80 days at 20 °C; nevertheless, our half-life was 241 days for β-carotene in SDE. Similarly, goji berry carotenoids extracted in sunflower oil halved in 100 days at 5 °C and 48 days at 25 °C, while in soybean oil the half-lives were 295 and 67 days, respectively [38]. In a further experiment, the carotenoids extracted from pumpkin halved in 142–301 d during storage at 25 °C and registered an average retention of 54.3–75.0% after 90 d [31]. Comparing the half-lives of carotenes in our encapsulates (Table 1), it is possible to observe that in almost all the cases the FDE encapsulates had higher values than the SDE, suggesting that the latter had a higher rate of degradation and confirming that freeze-dried encapsulates are more resistant to oxidation [12]. However, an adverse effect of freeze-dried encapsulate porous structure on stability of encapsulated bioactive compounds is also reported [39]. On the other hand, the spray-dried encapsulates have a lower water activity, which is beneficial in many aspects, such as for microbiological and chemical stability. Lavelli and Sereikaite [39] concluded that, when comparing spray- and freeze-dried encapsulates, water activity and core material play a detrimental role. The degradation rate also depends on the encapsulation efficiency and the amount of surface carotenoids which remain unprotected. Desobry et al. [40] reported a slightly higher percentage of surface carotenoids (38%) in spray-dried samples compared to freeze-dried samples (35%). In their study, the spray-dried β-carotene showed faster degradation kinetics than the freeze-dried one, due to their smaller particle size and higher surface carotenoid content.

The kinetics at 4 °C for all compounds and the kinetics of cis-β-carotene at all temperatures had low regression coefficients suggesting to exclude them from further analysis. Comparing the slopes of the regression lines of FDE and SDE at the same temperature (Figure 1), the t-test showed that in some cases freeze-drying was more effective (*p* ≤ 0.05) than spray-drying against carotenes degradation, such as for α- and β-carotene at 30 and 21.3 °C. An initial lag-time with no or very limited carotenes degradation was observed, ranging from 1 week at 37 °C up to 3 months (SDE) or more (FDE) at 21 °C. 

**Figure 2:**
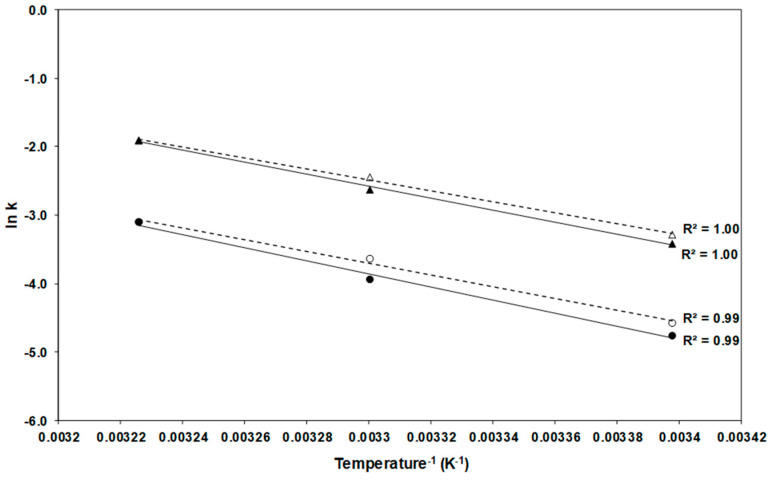
Arrhenius plot for α-carotene (●, ○) and β-carotene (▲, Δ) degradation during storage of freeze-dried (solid marks and solid regression lines) and spray-dried (hollow marks and dotted regression lines) encapsulated carrot-waste extract.

The Arrhenius plot (Figure 2) illustrates the effect of temperature on the degradation rate constants: the absolute value of the slope of the line is directly proportional to the activation energy (Ea) whose higher value implies closer relationship between variation of temperature and degradation [28].

Table 2 shows the Ea, which varied between 66.6 and 79.5 kJ/mol; the values tended to be higher in FDE than in SDE (Table 2). Similar results (66 kJ/mol) are reported for the degradation of total carotenoids in carrot slices blanched, dehydrated, and stored between 27 and 57 °C [41]. The Arrhenius model has been used by many authors to determine the stability of encapsulated carotenoids, but other models are found in literature, such as Weibull model, Regression model, Higuchi model, Hixson-Crowell cube root law, and Korsmeyer Peppas [12].

Our values on average were higher than the Ea observed in carotenes (range: 23.01–73.35 kJ/mol) by several Authors [27,28,35,36,37]. Generally, k, t_1/2_ and Ea depend on the source of carotenoids, the core material, and the storage conditions. During storage the carotenoids can form complex structures with a matrix which influence Ea. Since the FDE samples were found to be more stable, slower release of carotenoids, higher Ea and z values of both carotenes are reasonable; additionally, the concentration of carotenoids in encapsulates is also an important factor [12]. 

## 3. Materials and Methods

### 3.1. Reagents and Solvents

The chemicals used were β-carotene standard (Sigma, St. Louis, MO, USA); pyrogallol (99%; TCI, Tokyo Chemical Industry Co., Ltd., Tokyo, Japan); sodium chloride, potassium hydroxide (VWR International bvba, Leuven, Belgium); *n*-hexane (97% HPLC grade), tetrahydrofuran (99.7% HPLC grade; Avantor Performance Materials Poland S.A., Gliwice, Poland); ethyl acetate (99.7% HPLC grade; Sigma-Aldrich Co Chemie GmbH, Steinheim, Germany); ethanol (96%), methanol (HPLC grade), dichlorometane (stabilized with ethanol 0.1%; VWR International S.A.S., Fontenary-sous-Bois, France); butylated hydroxytoluene (Sigma-Aldrich Co., St. Louis, MO, USA).

### 3.2. Samples

The carrot waste was obtained from the “Nectar” beverage industry (Bačka Palanka, Serbia). The waste was immediately packed, freeze-dried, and stored at −20 °C until use. The sunflower oil used for carotenoids extraction, purchased from a local supermarket, was produced by the edible oil manufacturing company “Dijamant” (Zrenjanin, Serbia). The whey protein concentrate was purchased from Olimp Laboratories (Debica, Poland). The inulin was provided by Elephant Pharma (Belgrade, Serbia).

### 3.3. Carrot Waste Extraction and Encapsulates Preparation

Freeze-dried carrot waste was mixed with sunflower oil (1:10 *w/v*) at 25 °C by stirring with a B800E high-speed blender for 30 min (Gorenje, Velenje, Slovenia), using time shifts of 10 min blend and 5 min pause to avoid heating. The mix was then centrifuged at 2470× *g* for 10 min (centrifuge Lace 24, Colo Lab Experts, Novo Mesto, Slovenia), the supernatant was recovered and stored at 4 °C in a dark glass bottle, wrapped in foil, for further use.

The enriched oil was encapsulated by two different techniques, i.e., freeze-drying and spray-drying, according to the optimal conditions determined in a preliminary study [15]. The optimal wall materials (100% whey protein for freeze-drying; 71% whey protein and 29% inulin for spray-drying) were prepared as follows: for freeze-drying, the wall material was mixed with distilled water at 60 °C in a 1:2 (*w/v*) ratio and stirred until the temperature reached 30 °C; for spray-drying, the wall material was mixed with distilled water in a 1:8 (*w/v*) ratio following the same procedure. The wall material-oil extract solutions (100 g/60 mL), with the addition of the emulsifier Tween 80, were homogenized at 11,000 rpm by an Ultra-Turrax^®^ T25 (Ika-Labortechnik, Staufen, Germany) for 3 min at room temperature. The first formulation was kept overnight at −80 °C and then freeze-dried at −40 °C for 48 h with a Christ Alpha 2–4 LSC (Martin Christ, Osterode am Harz, Germany), to ensure complete drying; the freeze-dried encapsulates (FDE) were stored at −20 °C until further use. The second formulation was spray-dried using a Büchi mini B-290 (Büchi Labortechnik, Flawil, Switzerland) at an inlet temperature of 130 °C and an outlet temperature of 65 ± 2 °C. The spraying air flow and liquid feed rate were 600 L/h and 8 mL/min, respectively. The spray-dried encapsulates (SDE) were stored at −20 °C until further use.

### 3.4. Storage

The encapsulates were placed in two half-filled 50 mL screw-capped tubes per sample, stored in the dark at different temperatures: +4 °C, +21.3 °C (model FTX700, Cavallo, Buccinasco, Italy), +30 °C (model ICT 52Lt, FALC Instruments, Treviglio, Italy), +37 °C (model B 6060, Heraeus, Hanau, Germany) and analysed for their carotenoids content after different storage times up to 413, 413, 200, and 143 days, respectively.

### 3.5. Chemical Analysis

Carotenoids quantification was performed by reverse phase HPLC [13]. Briefly, 0.5 g of encapsulated were exactly weighted in a 25 mL screw-capped tube, wrapped in aluminum foil, and saponified under nitrogen for 45 min at 70 °C in thermostatic bath (Haake E8, Berlin, Germany), with the addition of 2.5 mL of ethanolic pyrogallol (60 g/L) as antioxidant, 1 mL of ethanol (95%), 1 mL of sodium chloride (10 g/L) and 1 mL of potassium hydroxide (600 g/L). During the saponification, the tubes were vortexed every 10 min. Afterwards, they were cooled in an ice bath and 7.5 mL of sodium chloride (10 g/L) were added. The suspension was then extracted twice with 15 mL of hexane:ethyl acetate (9:1 *v/v*). The organic layer was collected and evaporated under vacuum, followed by nitrogen drying; the residue was dissolved in 2 mL methanol:dichlorometane (1:1 *v/v*) and filtered through a 0.45 μm PTFE membrane. The filtered solution (20 μL) was injected in a HPLC system including: column Vydac 201TP54 C18, 250 × 4.6 mm, 5 μm (The Separation Group, Inc., Hesperia, CA, USA); LiCHrospher WP 300 RP-18, 5 μm (Merck, Darmstadt, Germany); mobile phase, methanol:tetrahydrofuran stabilized with 0.1% butylated hydroxytoluene (95:5, *v/v*); flow rate, 1 mL/min; pump Waters 510 (Millipore, Milford, MA, USA). Carotenoids were detected at 445 nm, using a Waters 996 series photodiode array detector (Millipore, Milford, MA, USA), controlled by the software Millenium 32 Chromatography Manager (Waters Chromatography Division, Millipore, Milford, MA, USA). The wavelength range was 200–600 nm. The quantification of the peaks was performed with the external standard method by preparing the calibration curve of β-carotene for all the compounds. The results are expressed in mg/kg dry matter (DM).

### 3.6. Degradation Kinetics Modelling

To determine the degradation reaction order of each carotenoid, both zero- and first-order kinetics were hypothesized by applying the general reaction rate expression $\frac{-dC}{dt}=kC^{n}$, where *C* is the concentration of the compound (mg/kg DM), *k* is the reaction rate constant, *t* is the reaction time (days) and *n* is the order of the reaction [42]. The order with the best correlation (r) and the best correspondence among the experimental values and the half-life of the compound (*t*_1/2_) [time for the concentration of a reactant to fall to half its initial value; $t_{1/2}=\frac{C_{0}}{2k}$ for zero order where *C*_0_ is the initial concentration, and $t_{1/2}=\frac{ln2}{k}$ for first order)] was selected. The temperature dependence of the reaction rate was determined by the Arrhenius equation, $k=Ae^{\frac{-E_{a}}{RT}}$, where *Ea* is the activation energy of the reaction (kJ/mol), *A* is the pre-exponential factor, *R* is the gas constant (8.314 J/mol·K) and *T* is the absolute temperature (K). The *z* value $\left(z=2.303\frac{RT^{2}}{E_{a}}\right)$ represents the increase in temperature that leads to a 10-fold increase in the reaction rate.

### 3.7. Statistical Analysis

The average values, standard deviations, linear regression, and kinetic constants were computed using the Microsoft Excel 2016 software (Microsoft, Redmond, WA, USA). The comparison between the slopes of the regression lines was performed with the STATGRAPHICS^®^ Centurion XVI v16.2.04 statistical software (Statpoint Technologies Inc., Warrenton, VA, USA).

## 4. Conclusions

In encapsulated carrot waste extracts, the carotenoids degradation rate was dependent on the temperature, following a zero-order kinetics, while at 4 °C the carotenes were stable for at least 413 days. The freeze-drying technique was more effective against the degradation of α- and β-carotene, particularly at 30 and 21.3 °C. An initial lag-time with no or very limited carotenes degradation was noticed and spanned from one week at 37 °C up to 3 months (SDE) or more (FDE) at 21 °C. These findings suggest that encapsulation of carrot waste extract by freeze-drying and spray-drying, using as wall materials whey protein and whey protein-inulin blend, respectively, ensures good carotenes protection during storage. In conclusion, the encapsulates of carrot waste oil extract showed a good shelf life even at room temperature, suggesting their use as a promising ingredient for the preparation of food products. Further studies might be needed to evaluate and optimize their shelf life performance under different packaging materials and conditions. 

## Figures and Tables

**Table 1:** Reaction rate constants (*k*; mg/kg DM·days for zero-order; 1/days for first-order) and half-lives (*t*_1/2_; days) for zero-order carotenoids degradation during storage of freeze-dried (FDE) and spray-dried (SDE) encapsulates of carrot-waste extracts.

		α-Carotene			β-Carotene			*cis*-β-Carotene		
	°C	*r*	*k*	*t*_1/2_	*r*	*k*	*t*_1/2_	*r*	*k*	*t*_1/2_
*Zero-order*										
FDE	4	0.21	0.000		0.55	0.000		0.41	0.001	
	21	0.98	0.009	319.1	0.98	0.033	281.2	0.81	0.007	366.7
	30	0.99	0.019	141.1	0.98	0.071	129.2	0.95	0.019	130.8
	37	0.99	0.045	61.3	1.00	0.149	61.9	0.89	0.058	43.1
SDE	4	0.56	0.001		0.44	0.000		0.78	0.004	
	21	0.99	0.010	265.2	0.99	0.037	241.3	0.99	0.013	256.7
	30	1.00	0.026	103.9	0.99	0.087	104.2	0.98	0.036	94.7
	37	0.99	0.045	60.7	1.00	0.148	61.1	0.94	0.064	53.1
*First-order*										
FDE	4	0.20	0.000		0.54	0.000		0.39	0.000	
	21	0.96	0.003		0.94	0.003		0.88	0.002	
	30	0.97	0.006		0.96	0.007		0.93	0.006	
	37	0.87	0.031		0.88	0.029		0.91	0.029	
SDE	4	0.55	0.000		0.43	0.000		0.76	0.001	
	21	0.95	0.004		0.93	0.004		0.94	0.004	
	30	0.92	0.014		0.91	0.014		0.90	0.014	
	37	0.93	0.026		0.91	0.029		0.91	0.027	

**Table 2:** Activation energies (Ea) and z for carotenoids degradation during storage of freeze-dried (FDE) and spray-dried (SDE) encapsulates of carrot-waste extracts.

	Ea (kJ/mol)	z (°C)	Ea (kJ/mol)	z (°C)
	FDE		SDE	
α-carotene	79.2	22.11	71.7	24.42
β-carotene	72.8	24.05	66.6	26.30

## Data Availability

The data presented in this study are available on request from the corresponding author.

## Sample Availability

Samples of the encapsulates are available from the authors.
